# Theoretical and Experimental Research on the Short-Range Structure in Gallium Melts Based on the Wulff Cluster Model

**DOI:** 10.3390/ma18010133

**Published:** 2024-12-31

**Authors:** Chun Wang, Minghao Hua, Luyao Wang, Shenglong Wang, Jinlong Liu, Rong Liu, Xuelei Tian, Xiaohang Lin

**Affiliations:** Key Laboratory for Liquid-Solid Structural Evolution and Processing of Materials, Ministry of Education, Shandong University, Jinan 250061, China; 202314148@mail.sdu.edu.cn (C.W.); huaminghao@outlook.com (M.H.); 202334253@mail.sdu.edu.cn (L.W.); 202234232@mail.sdu.edu.cn (S.W.); 202214130@mail.sdu.edu.cn (J.L.); 202414167@mail.sdu.edu.cn (R.L.); tianxuelei@sdu.edu.cn (X.T.)

**Keywords:** gallium melts, Wulff cluster model, short-range ordering, high-temperature XRD, DFT, surface energy

## Abstract

In this paper, the short-range ordering structures of Ga melts has been investigated using the Wulff cluster model (WCM). The structures with a Wulff shape outside and crystal symmetry inside have been derived as the equivalent system to describe the short-range-order (SRO) distribution of the Ga melts. It is observed that the simulated HTXRD patterns of the Ga WCM are in excellent agreement with the experimental data at various temperatures (523 K, 623 K, and 723 K). This agreement includes first and second peak positions, widths, and relative intensities of patterns, particularly at temperatures significantly above the melting point. A minor deviation in the second peak position has been observed at 523 K, attributed to the starting of the pre-nucleation stage. These findings demonstrate that the WCM can effectively describe the SRO structure in melt systems exhibiting a certain extent of covalency.

## 1. Introduction

In recent years, gallium and its alloys have played an important role in many fields such as semiconductor devices; solar cells; catalysis [1,2,3,4,5,6,7,8], especially for the manufacture of microwave communication and microwave integration; infrared optics and infrared detector devices; integrated circuits; and light-emitting diodes, etc. [9,10]. To enhance the quality and improve the gallium production manufacturing techniques, a deep understanding of the short-range structures of Ga melts is necessary. This is the key to the properties of the melt and subsequent products.

To investigate the melt structure, experimental methods such as HTXRD [11], X-ray absorption fine structure (EXAFS) [12,13], neutron scattering [14], and so on, are widely used. Intensity patterns and pair distribution functions (PDFs) have been intensively studied through intensity correction and Fourier transformation [15,16,17], although the fundamental physical picture of the SRO of the melts is obscured by the high-temperature and liquid experimental conditions. In other words, the physical definition of the liquid state remains incomplete. In this case, simplified physical models such as the hard sphere model, microcrystalline model, and cluster model [18,19,20,21] have been built to help researchers understand the experimental phenomena. However, determining the atomic-scale structure of metallic melts is still challenging. This is intertwined with several unresolved issues, such as the non-Arrhenius changes in viscosity and the amorphous SRO structures.

In prior research, a thermodynamic statistical model, namely WCM, was built by our group with the aim of describing the SRO of the melts. In this model, the most probable microstructure is regarded as an equivalent system of SRO in melts within a state of thermodynamic equilibrium. The most probable microstructure is given by the Wulff shape within the corresponding crystalline structure. Meanwhile, the size of the microstructure is derived directly from the experimental data obtained via HTXRD. This model is successfully applied to melts of pure metals [22], binary homogeneous alloys [23], eutectic alloys [24], and alloys with intermetallic compounds [25]. All of the above systems have a common feature, namely, that the bonding within them is a metallic bond. The covalent bond system (like Ga) is a kind of system that has never been studied in this way. The widespread use of semiconductors makes the study of the melt structure within a covalent bond system very necessary because it is still unclear.

In this article, the melt SRO of a system with covalent bonds (Ga) is investigated using the Wulff cluster model under thermodynamic equilibrium. The suitability of WCM in the system with covalent bonds has been proven by HTXRD experiments.

## 2. Methods

### 2.1. Experimental Methods

The pure gallium specimen employed in this research consisted of 15 g of pure gallium with a purity of 99.99%. Prior to the measurement, we ensured that there was no oxide on the specimen. Subsequently, the molten specimen was analyzed using HTXRD(Jinan, China) with a Mo target serving as the X-ray source. The sample was superheated at 773 K for 50 min within an yttria crucible, and then gradually cooled to the target temperatures (723 K, 623 K, and 523 K). Before the liquid X-ray diffraction patterns were measured, the samples were maintained at the respective temperatures for 30 min. The specific parameters were as follows: Generator Current was set at 40 mA, Generator Voltage was 45 KV, and the scanning angle (2θ) was from 10° to 80° with 0.5° step length. The exposure time for each step was 30 s. After atomic scattering correction and a nonlinear transformation of the intensity curve, the structure factor curve S(Q) can be derived using the following formula [25]:

(1) $$S(Q)=\frac{I(\theta )}{N{f(Q)}^{2}}$$

Here, f(Q) represents the atomic form factor, which is obtained from the International Tables for Crystallography [26]; N is defined as the number of atoms engaged in diffraction; Q, which is a nonlinear scaling of θ, is the magnitude of inverted lattice vector, computed as Q=4πsinθ/λ, where λ designates the X-ray wavelength employed in the HTXRD measurement. The PDF ρ(r) is employed to depict the number density of atoms on a spherical shell centered around an atom with a radius r from the center. It constitutes a fundamental approach for probing the distribution of atoms within the molten state. S(Q) could be described by the formula as follows [25]:

(2) $$S(Q)=1+\int_{0}^{\infty }4\pi r^{2}[\rho (r)-\rho_{0}]\frac{sin(Qr)}{Qr}dr$$

Here, $\rho_{0}$ represents the average density determined by macroscopic density and atomic mass. Although the utilization of certain auxiliary functions and mathematical approximation methods emerged, the PDF ρ(r) could be acquired by sinusoidal transformation. Experimentally [27,28], the correlation radius $R_{c}$, according to Formula (3), is regarded as the size of the equivalent structure [25].

(3) $$\left|\frac{\rho (r)}{\rho_{0}}-1\right|<0.02\;\;\;(r>R_{c})$$

### 2.2. Simulation Methods

In the present article, the Vienna ab initio Simulation Package 6.3 (VASP 6.3) is utilized for first principle calculation with the aim of obtaining the surface energy γ. The generalized gradient approximation (GGA) exchange-correlation potential and the Perdew–Burke–Ernzerhof (PBE) exchange-correlation function are chosen [29,30]. The projector augmented wave (PAW) pseudopotential potentials constructed by Kresse and Joubert is used [31,32]. After conducting convergence tests, an energy cutoff of 500 eV is selected. The Monkhorst–Pack scheme with 7 × 7 × 1 k-points is selected to ensure a satisfactory convergence of the energy for slab modeling. Concurrently, the alternative Monkhorst–Pack scheme with 7 × 7 × 7 k-points is deemed an appropriate selection for pure gallium bulk modeling (as will be detailed subsequently). The relaxation of most stable structures continues until the residual forces of the total structure are less than 0.01 eV/Å. In prior research, it has been established that the relative proportions of distinct faces in the Wulff shape, as determined by interface energy, closely approximate those computed via γ [23,33,34]. Therefore, it is reasonable to use the computed γ instead of interface energy to describe the structure of the Wulff model.

The influence of temperature on various surfaces has been meticulously investigated. It has been proved that a finite temperature hardly influences the γ. Specifically, when compared with the surface energy at 0 K, the discrepancy in surface energy remains less than 0.5% even at 800 K [21,22,23,24]. Consequently, γ at 0 K can be adopted for the construction of the Wulff shape.

**Figure 1:**
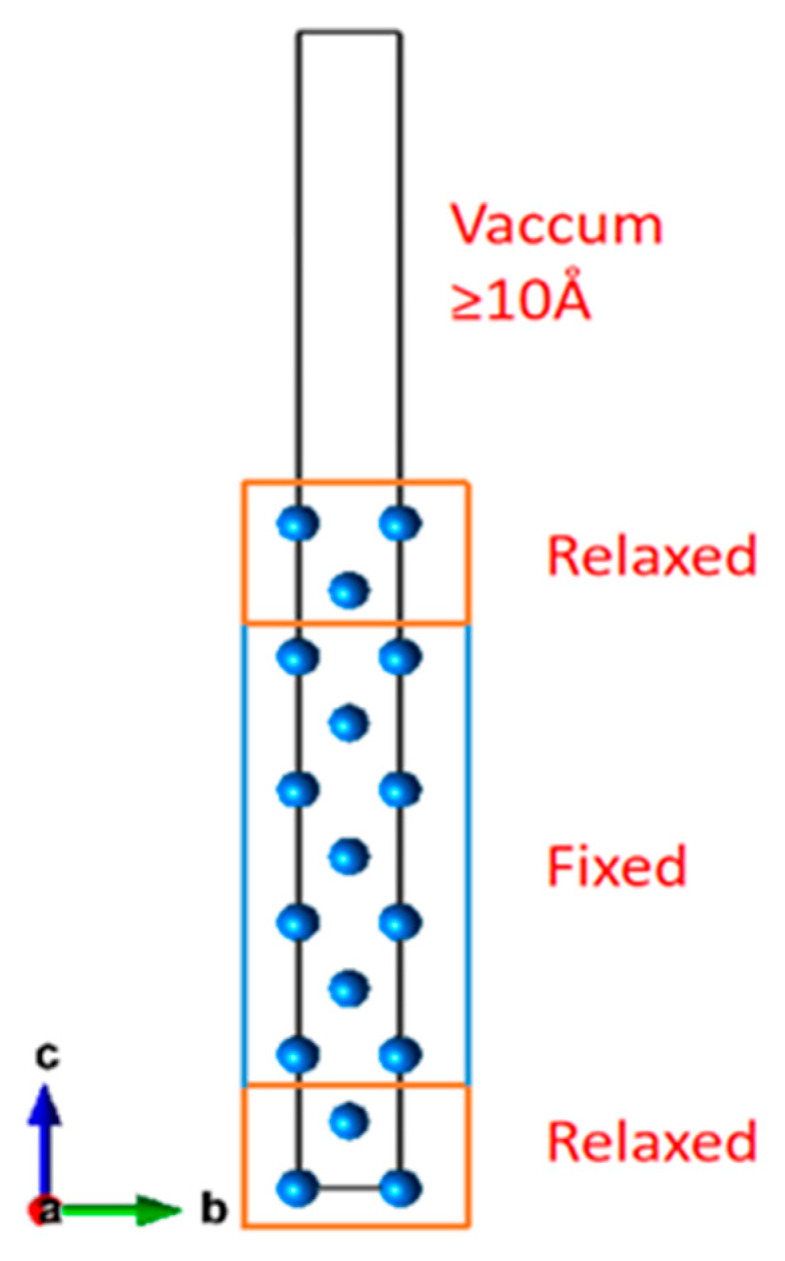
Demonstration of the double-faced slab model.

For the purpose of calculating the surface energy of different crystallographic plane families, the double-faced slab model, which is shown in Figure 1, is employed. In this model, specific atoms are extracted from a bulk supercell, thereby creating a vacuum layer and simultaneously exposing the desired crystal plane. One lattice vector c of the supercell is arranged to be perpendicular to the exposed surface, whereas the other two lattice vectors are configured to be parallel to it. The atoms located in the vicinity of the center of the slab model, which are considered the atoms belonging to the bulk phase, are fixed (ordinarily approximately 60%). In contrast, the outermost atoms, which are representative of the surface atoms, are permitted to relax. The surface energy, denoted as γ, is determined by [35]:

(4) $$\gamma =\frac{1}{2A}(E_{salb}-{NE}_{bulk})$$

In Equation (4), A designates the area of the crystallographic surface that is exposed within the model. $E_{salb}$ represents the total energy of the system, $E_{bulk}$ corresponds to the total energy of the primitive cell in the bulk phase (bulk model), while N signifies the quantity of primitive cells.

To enable a comparison with the experiment data, the X-ray diffraction (XRD) pattern is simulated. This is achieved by inversely solving the Laue equation using the aforementioned cluster. The angle step length is configured to be 0.05°, and the full width at half maxima (FWHM) is configured to be smaller than the step length, thereby facilitating the acquisition of peak position. In the output data, the peaks with intensities less than 10% of the maximum peak intensity are excluded. Subsequently, the residual peaks are expanded in accordance with the Gaussian peak function to mimic the small-size effect and the significant lattice distortion of the atomic cluster structure within a melt. A temperature background function is incorporated to simulate the alterations in the X-ray intensity distribution that result from temperature variations. The ultimate broadening formula is as follows [36]:

(5) $$I\left(2\theta \right)=\sum_{i=1}^{n}\left\{I_{i}\left[\frac{P_{1}}{\sqrt{2\pi }\cdot \sqrt{a_{1}^{2}+b^{2}}}e^{-\frac{{\left(2\theta -2\theta_{i}\right)}^{2}}{2\left(a_{1}^{2}+b^{2}\right)}}+\frac{P_{2}}{\sqrt{2\pi }\cdot \sqrt{a_{2}^{2}+b^{2}}}e^{-\frac{{\left(2\theta -2\theta_{i}\right)}^{2}}{2\left(a_{2}^{2}+b^{2}\right)}}\right]\right\}$$

where $I_{i}$ is associated with the intensity of simulation peaks; $P_{1}$ denotes the proportion of the number of inner atoms to the total amount of atoms within the WCM; $P_{2}$ represents the ratio of outer atoms; $a_{1}$ and $a_{2}$ relate to the peak widening induced by the inner and outer lattice distortions, respectively; and *b* is related to the peak widening caused by fine crystallization. The parameters were ascertained based on experimental observations and were suitably adjusted within an appropriate range.

## 3. Results and Discussion

### 3.1. HTXRD Results of Pure Gallium

**Figure 2:**
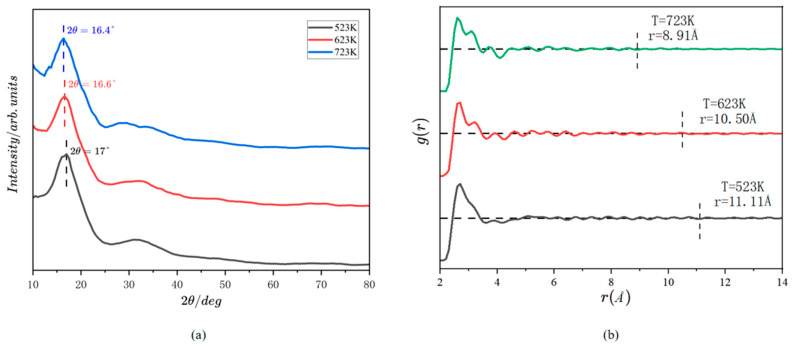
(**a**) XRD intensity pattern and (**b**) PDF of the pure gallium melt at temperatures of 523 K, 623 K, and 723 K.

The HTXRD patterns of the pure Ga samples at temperatures of 523 K, 623 K, and 723 K are displayed in Figure 2a. The discrepancies among the intensity curves under these three temperatures are rather subtle, and the positions of the most prominent peaks do not exhibit significant alterations. The peaks are attributed to the SRO structures (clusters) within the melts, while the background height is a consequence of the free atoms scattering (disordered atoms). It can be found that the strongest peak of each curve shifts to the right with a very small amplitude as the temperature shifts from high to low, which appears at 16.4° (723 K), 16.6° (623 K), and 17° (523 K). At the same time, the position of the secondary peak moves right slightly with the reduction in temperature, which appears near 32° and has a wider peak shape compared with the first peak. In addition, the first peak is about four times stronger than the second one. To obtain the pair distribution function, the temperature background ought to be removed from the experimental data; then, the data are normalized and transformed into S(Q) through Formula (1). S(Q) undergoes sinusoidal transformation to ρ(r) by Formula (2). Subsequently, it is divided by the PDF $\rho_{0}$ to obtain g(r), shown in Figure 2b. From Formula (3), the average correlation radii of the melts at 523 K, 623 K, and 723 K are 11.11 Å, 10.50 Å, and 8.91 Å, respectively. These radii exhibit a tendency to increase with the decrease in temperature, which is in consonance with the prior anticipation.

### 3.2. Basic Characteristics of Gallium

**Figure 3:**
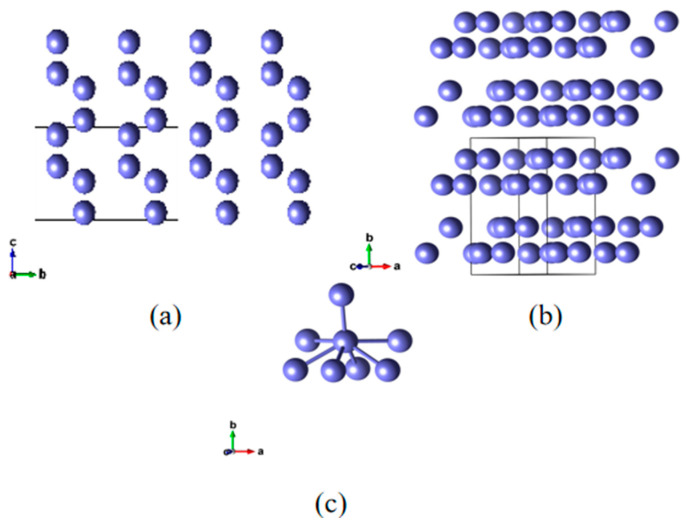
(**a**,**b**) Pure gallium bulk structure in two perspectives. (**c**) Gallium atom coordination relationship.

Gallium is a kind of special metal that has certain extent covalency in the crystal state. All of the computational structures are founded upon α-Ga, which adopts an orthorhombic crystal system with four atoms residing within the primitive cell. Each atom is in proximity to seven neighboring atoms, thereby giving rise to a greatly anisotropic atomic environment, and it is the single crystal phase of gallium that can exist chronically under normal pressure and temperature [37,38,39,40,41,42]. The specific structure is shown in Figure 3.

Compared with the experiment data [43], the gallium crystal lattice parameters shown in Table 1 have a maximum error of only 3%, which indicates that the chosen model and parameters are reasonable.

In Table 1, A, B, and C represent lattice constants. Each Ga atom has one very close neighboring atom and six atoms only a little further away, which is shown in the table.

**Figure 4:**
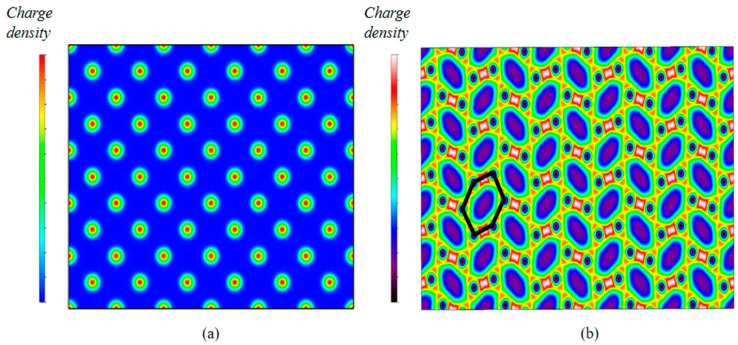
(**a**) The charge density distribution of the Cu bulk from the view of Cu (100) direction. (**b**) The charge density distribution of the Ga bulk from the view of Ga (100) direction.

The charge density distributions of Cu and Ga are shown in Figure 4. The red part in the color axis denotes the region of high charge density, and the blue and black parts in the color axis represent the region of low charge density. Generally speaking, the valence electrons of typical metals (like Cu) exhibit nonlocalized characteristics. In Figure 4a, the blue area indicates the uniform charge density in the Cu bulk. Unlike typical metals, the valence electrons are more localized between the Ga atoms (area in the figure consisting of a hexagonal ring marked in the figure), which shows in the red color in Figure 4b. The other areas have relatively low charge densities between the rings of six Ga atoms (the area surrounded by the black line). It is obvious that the bonds exhibit the properties of covalent bonds in the Ga crystal.

### 3.3. Wulff Cluster Model of Ga

The HTXRD experiments mentioned above were repeated serval times at each temperature with extremely consistent results, which obviously demonstrates the melts are a thermodynamic equilibrium system. On the one hand, according to the Wulff construction, the most probable shape should have the lowest total surface Gibbs free energy, determined by the interface energies [35]. On the other hand, the electron distribution, which should obey Fermi–Dirac distribution, determines many properties of the crystals, especially the point group symmetry. The electron distribution hardly changes significantly until the temperature reaches the Fermi degeneracy temperature (FDT). Considering that the FDT typically exceeds in the order of magnitude of 10^4^ K, the temperature of metallic crystals and melts (generally in the order of magnitude between 10^2^ K and 10^3^ K) are considerably low. As a result, the trend in point group symmetry hardly changes between these two phases. Hence, the structures with a Wulff shape outside and crystal symmetry inside have been derived as the equal system for characterizing the SRO distribution within the microstructure of the melts.

**Figure 5:**
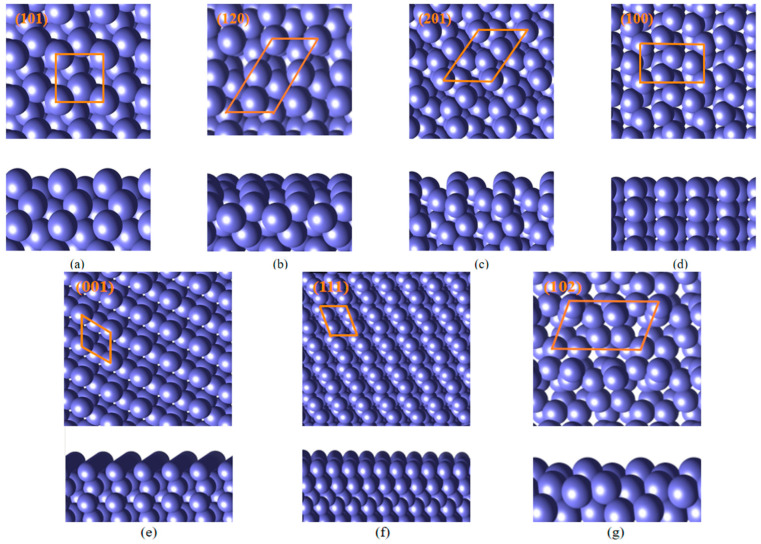
Top and side views of the (**a**) Ga (101) surface, (**b**) Ga (120) surface, (**c**) Ga (201) surface, (**d**) Ga (100) surface, (**e**) Ga (001) surface, (**f**) Ga (111) surface, (**g**) Ga (102) surface. The area surrounded by the orange line in the figure is the unit of the surface. All surfaces that are exposed on the Wulff shape are shown in the figure.

Although the Wulff cluster model that has the lowest total surface Gibbs free energy on the cluster’s surface could describe all kinds of metals in principle, the applicability in the crystal with the properties of covalent bonds still needs investigation. To determine the Wulff shape of Ga, surface energies of Ga surfaces are calculated using the two-side slab model mentioned above. The result of the calculation determines which crystal planes will be exposed on the cluster surface. The established slab model is shown in Figure 5. Low Miller index crystal planes with low surface energies are selected to establish the slab modeling and compute the γ. The specific data of γ are displayed in Table 2. All of the data are relatively reasonable compared with other DFT calculation results [44].

**Figure 6:**
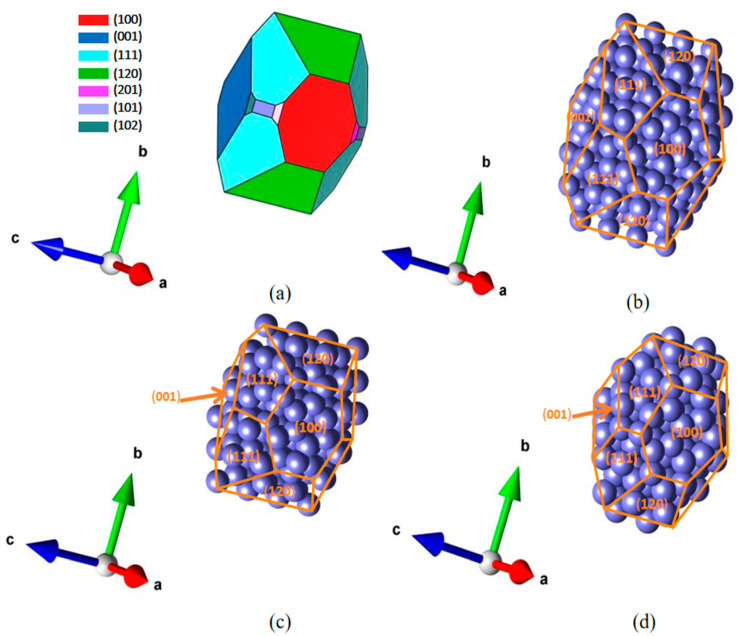
(**a**) The schematic of the Ga Wulff shape. The atomic equivalent structure of Ga melts at (**b**) 523 K, (**c**) 623 K, and (**d**) 723 K.

As shown in Table 2, the Ga (100) plane, among all crystalline planes calculated, has the minimum γ (0.477 J/m^2^). During the calculation process, we found that the distance between the nearest neighboring atoms on the (100) surface decreased and dimerization occurred, which is the main reason for the lowest γ. For the low-index surfaces of Ga that are calculated, the trend of surface energy $\gamma_{100}$ < $\gamma_{001}$ < $\gamma_{101}$ is apparent. These results indicate that the density of each crystal plane also has an impact on surface energy, and the (100) plane is precisely the most close-packed plane of Ga. Meanwhile, the relatively low-index crystal planes are prone to being exposed on the Wulff cluster. In addition, the crystal planes can be exposed more on the cluster when the surface energy γ of the plane decreases to a certain extent compared with the other planes. On the basis of the modeling and above-mentioned calculational results, the shape of WCM that is depicted according to the calculated γ data is displayed in Figure 6a. For the Ga cluster, the (100) surface’s ratio of corresponding exposed area is the largest (about 30.96%). The shape of the WCM of the Ga cluster is similar to a spindle shape.

To acquire the Wulff cluster, the average correlation radii and other related data mentioned above are given by the HTXRD experiments, and the atomic equivalent structures of the Ga melts are displayed in Figure 6b–d. It can be observed that for the Ga Wulff atomic microstructure, the majority of the calculated faces exist. Due to the small cluster size, relatively high index faces with relatively low surface energies, such as the Ga (210) and Ga (310) surfaces, cannot be exposed on the Wulff cluster.

**Figure 7:**
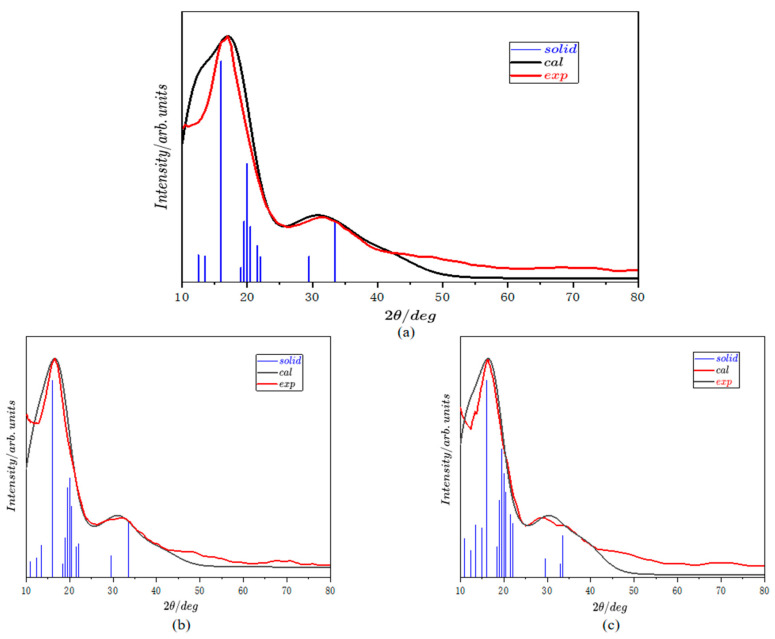
The XRD simulation results compared with the experimental data at (**a**) 523 K, (**b**) 623 K, and (**c**) 723 K.

To check the accuracy of WCM within the melts featuring covalent bonds, it is necessary to conduct a direct comparison between the calculated XRD results and the experiment data shown in Figure 7. The blue perpendicular lines denote the diffraction patterns of the atomic structures above at 0 K, the red curve is the experimental XRD results, and the black one is the calculated broadening XRD curve. Not only the positions but also the comparative intensities of the two peaks of the simulated XRD curves agree with the experimental data quite well, especially at high temperatures (623 K and 723 K). Note that a slight deviation of the position of the second peak at 523 K takes place. Such phenomena happen at low temperatures near the melting point and have been observed in almost all metallic alloy systems [19,21,22]. It is usually caused by the beginning of the pre-nucleation stage. Determining the short-range structure of gallium melts serves as a foundation for further research into gallium-based liquid alloys, providing us the opportunity to understand the inner interactions within these materials. This gives us the chance to design the composition and processing techniques of gallium-based liquid metals with an industrial product-oriented approach, which will significantly promote the development of industries such as lubrication and flexible electrodes.

## 4. Conclusions

In this paper, the SRO structures of Ga melts has been investigated by the WCM, which has been proven to successfully describe the melt structure of pure metals, homogenous alloys, eutectic alloys, and alloys with intermetallic compounds. Structures with Wulff shapes outside and crystallographic symmetry inside have been derived as the equivalent system to describe the SRO distribution of the Ga melts. The XRD curves of Ga melts at different temperatures (523 K, 623 K, 723 K) were obtained in the experiment, and the g(r) and the correlative radius r (11.11 Å, 10.50 Å, 8.91 Å) were obtained accordingly. The γ data of different crystal planes of Ga crystals were acquired in the DFT calculation, and the γ of the (100) plane was the lowest (0.477 J/m^2^). Based on the above results, the atomic equivalent structures at different temperatures have been presented. Through comparison with the experimental outcomes of HTXRD, it has been discovered that overlaying the HTXRD pattern of the gallium WCM enables the simulated HTXRD pattern to be in excellent agreement with the experimental data at the designated temperatures. This includes aspects such as the peak position, width, and relative intensity, particularly at temperatures significantly above the melting point. A tiny deviation of the second peak position at 523 K occurred, which was caused by the beginning of the pre-nucleation stage. In this case, the WCM can describe the SRO structure of melt systems with a certain extent of covalency, such as Ga.

## Figures and Tables

**Table 1:** Lattice parameter (Å) of the Ga bulk.

Parameter	A (Å)	B (Å)	C (Å)	Nearest Neighboring (Å)	Second Nearest Neighboring (Å)
Experiment	4.51	4.49	7.65	2.44	2.71–2.79
Calculation	4.63	4.55	7.77	2.51	2.73–2.77

**Table 2:** Surface energy calculation of Ga cluster.

Crystal Plane	Surface Energy γ (J/m^2^)	Percentage (%)	Crystal Plane	Surface Energy γ (J/m^2^)	Percentage (%)
Ga (001)	0.591	21.670	Ga (102)	0.643	0.690
Ga (100)	0.477	30.960	Ga (201)	0.590	0.770
Ga (101)	0.618	1.380	Ga (211)	0.605	0.000
Ga (111)	0.592	18.640	Ga (310)	0.554	0.000
Ga (120)	0.607	25.960	Ga (010)	0.847	0.000

## Data Availability

The data that support the findings of this study are available within the article.
